# Socio demographic profile and utilization pattern of antipsychotic drugs among schizophrenic inpatients: a cross sectional study from western region of Nepal

**DOI:** 10.1186/1471-244X-13-96

**Published:** 2013-03-22

**Authors:** Indrajit Banerjee, Bedanta Roy, Brijesh Sathian, Indraneel Banerjee, Prasanta Kumar Chakraborty, Archana Saha

**Affiliations:** 1 Department of Pharmacology, Manipal College of Medical Sciences, Pokhara, Nepal; 2 Department of Physiology, Manipal College of Medical Sciences, Pokhara, Nepal; 3 Department of Community Medicine, Manipal College of Medical Sciences, Pokhara, Nepal; 4 R. G. Kar Medical College, Kolkata, West Bengal, India; 5 Manipal Teaching Hospital, Pokhara, Nepal; 6 Department of Pharmacology, Manipal College of Medical Sciences, Pokhara, Nepal

**Keywords:** Antipsychotics, Drug utilization study, Nepal, Psychiatry, Schizophrenia

## Abstract

**Background:**

Currently a large number of atypical antipsychotics available in the market are endorsed as better option for treating schizophrenia than the typical antipsychotics. Information regarding the utilization pattern of antipsychotic drugs is lacking in Nepalese population particularly in Western Nepal. By means of this study one is expected to acquire an idea concerning clinician’s preference to the antipsychotic drugs in actual clinical setup. The main objective of the study was to find the commonest antipsychotics prescribed in a tertiary care center among hospitalized patients in Western Nepal.

**Methods:**

This cross sectional study was carried out between 1st January 2009 and 31th December 2010 at Manipal Teaching Hospital, Nepal. The diagnosis of schizophrenia was based on ICD-10 (Tenth revision).The main outcome variables of the study was commonest antipsychotic drug prescribed. Z test, Chi square test and logistic regression were used for analytical purpose. P-value < 0.05 was considered to be statistically significant. This is the first study done on the utilization pattern of antipsychotics drugs among hospitalized patients in Nepal.

**Results:**

Out of 210 cases of schizophrenia, most of the patients were less than 40 yrs. 78.6%, male 61.9%, unemployed 86.7% and having their monthly income less than NPR 10000 /month 80.5%. As far as religion, 78.1% patients were the Hindus and ethnically schizophrenia was common among the Dalit 26.2%. The study revealed that 46.2% of patients were students followed by 25.2% of housewives. Olanzapine was the commonest antipsychotic drug to be prescribed 34.3%. It was observed that the psychiatrists had a tendency of using antipsychotic drugs by trade names [OR 3.3 (1.407, 8.031)] in male patients as compared to female patients.

**Conclusion:**

According to the utilization pattern of antipsychotics, it is concluded that atypical antipsychotics were used relatively more commonly than that of typical antipsychotics. Among the atypical antipsychotic drugs, there is a trend of using Olanzapine during Schizophrenia as compared to other atypical antipsychotic drugs in Western Nepal.

## Background

According to WHO, Drug utilization study is defined as a study of marketing, distribution, prescription and uses of drugs in a society highlighting on the resulting medical, social and economic consequences [1]. Medical aspects focus on the need to balance between the risk and the benefits. The benefits are assessed on the basis of drug efficacy in preventing, relieving and curing diseases or their symptoms and complications. Potential risks include short term and long term adverse effects involving special risk factors mainly associated with genetics, disease, environment, nutrition, age, sex, pregnancy, lactation. A social aspect primarily deals with impact of drugs in the society. It focuses on the attitudes of people towards drug use, and current trends of treatment verses insistent use of conventional medicines. The practice of drug abuse and subsequent causes are also essential social components. Economic aspect reflects on the prices and the applicable heath benefits of a given drug. This includes the drug prices, import verses local products and the costs of drug treatment verses non drug treatment. Current and future allocation of national resources to the drug and health budget is also an economic component [2]. Drug utilization research affords a baseline reference point about the effect of diverse interventions on prescribing the concerned drugs.

Globally the prevalence of schizophrenia shows a wide range of differences [3]. It is estimated that the median prevalence of schizophrenia is 4.6/1,000 for point prevalence, 3.3/1,000 for period prevalence, 4.0/1000 for lifetime prevalence and 7.2 for lifetime risk of morbidity across the world [4]. A study conducted by Robin RW et al. reports that globally the lifetime prevalence of schizophrenia is 1% [5]. According to standardized mortality ratio, the individuals with a complaint of schizophrenia have a 2-3-fold increased mortality risk as compared with the general population [6,7].

Evidently, there is a dearth need of relevant literatures concerning schizophrenia in Nepal as there is no adequate research about it. A recent study conducted at psychiatric clinic in Janakpur, Nepal showed high prevalence of schizophrenia (30%) [8]. Similar nature of result was found in a tertiary care mental hospital in Kathmandu, Nepal. The research finding stated schizophrenia (50.1%) as a common psychiatric disorder [9] whereas a study from Jiri, Eastern Nepal revealed low prevalence of schizophrenia (1.4%). Comparatively, this figure is quite small as compared to other psychiatric disorders [10]. Studies have shown that the outcome of schizophrenia is better in developing countries [11]. Following the recent evidences of a systemic review and Meta-analysis on antipsychotics among adults with schizophrenia, it has shown that the benefits of atypical antipsychotics over typical antipsychotics remain indecisive because of discrepancy in assessing the result [12]. A study carried out in Nepal indicates that both atypical and typical antipsychotics were effective to reduce the psychopathological symptoms of schizophrenia among Nepalese population. Interestingly, the finding of this study is contradictory to another study which revealed that atypical antipsychotics like Risperidone has quicker and better safety profile than typical antipsychotics like Haloperidol [13]. Nepalese population includes a wide variety of ethnic groups, to name a few, the Brahmin, Chettri, Gurung, Newar, Puns, Magar [14]. The level of knowledge and information with regard to the utilization pattern of antipsychotic drugs among Nepalese population is far less than present requirement. A 2001 study conducted by Shankar PR et al. at outpatient department of Psychiatry in Western Nepal confirms that the utilization pattern of antipsychotics was very low 8.6% as compared to other psychotropic drugs like antidepressants 45.94%, anxiolytics 19.41% [15].

The main objective of the study was to find out the commonest antipsychotics prescribed for patient with schizophrenia among hospitalized patients in western Nepal. The specific objectives were to find out whether the antipsychotics were prescribed by generic or trade names, essential or nonessential drugs, drug therapy or combination of drugs and psychotherapy, groups of antipsychotics prescribed, socio demographic details of schizophrenic patients in Nepalese context. This is the first study undertaken in the utilization pattern of antipsychotics among the hospitalized patients in Nepal.

## Methods

### Study design and the participants

The present study was a cross sectional study done at Manipal teaching hospital, Pokhara, Nepal. It is a tertiary care hospital of Western Nepal and has been providing services to patients from 1998 onwards. It was expected that all the critically ill psychiatric cases with schizophrenia will report at this hospital Western Nepal.

### Data collection

The study was carried out between 1^st^ October 2009 and 30^th^ September 2010 at Psychiatric ward in Manipal Teaching hospital. The collected data include socio demographic details such as age (<40 years and >40 years), gender (male and female), occupation (housewife, teacher, laborer, student, farmer, retired and others), religion (Hindu, Buddhist, Muslim), ethnicity (Brahmin, Chettri, Newar, Dalit and others), employment (employed and unemployed), monthly income NPR (<10000/month and >10000/month, groups of drugs (miscellaneous comprising of both typical and atypical antipsychotics combined together, atypical antipsychotics, typical antipsychotics) Treatment (ECT, drug therapy, drug and psychotherapy combined, based on essential drug list (essential or non-essential), trade/generic, commonest antipsychotic drug. Anatomical Therapeutic Chemical Code was also used for different antipsychotics prescribed [16].

### Inclusion criteria

The study conducted between 1st October 2009 - 30^th^ September 2010, includes 210 cases of critical ones with all types of schizophrenia but there was no age limit of the patients. The diagnosis of the disease was based on ICD-10 (Tenth revision) Classification of mental and behavioral disorders, Diagnostic Criteria for Research [17,18]. The total number of cases includes inpatient psychiatric cases and those were both inpatient and outpatient.

### Exclusion criteria

Out of 565 psychiatry inpatients, 355 patients were excluded following the diagnosis of psychiatric disorder as the study requires the participation of patients who are suffering from Schizophrenia only. Other additional cases, namely depression, anxiety, mania, bipolar disorder, substance abuse, suicidal tendencies and mental retardation were excluded from the study. The out patients were also excluded as the research aims to study about the drug utilization pattern among the patients who were critically ill with schizophrenia.

### Sample size calculation

For 95% confidence interval and, significance level α = 5%, P = 90%, Q = 10%, allowable error = 5%, required sample size was 171. P = percentage of antipsychotic drugs used for the treatment of Schizophrenia. In the pilot study done prior to the original study with 10 patients was admitted in the psychiatry ward with Schizophrenia [18].

### Outcome variable

The main outcome variable was the commonest antipsychotic drug prescribed.

### Explanatory variables

The demographic and psychiatric disorders have been defined at individual level. Factors which are taken into account at individual level were Age (<40 years and >40 years), gender (male and female), monthly income (<10000/month and >10000/month), employment of the patient (employed and unemployed), occupation (housewife, laborer, student, farmer, retired and others), religion (Hindu, Buddhist, Muslim), ethnicity (Brahmin, Chettri, Newar, Dalit and others).

### Ethical committee approval

Prior the study, ethical committee approval was taken from the institutional ethical committee, Manipal Teaching hospital, Pokhara, Nepal. The Research was conducted in accordance to latest version of the Declaration of Helsinki.

### Data management and statistical analysis

The data collected was analyzed using Excel 2003, R 2.8.0 Statistical Package for the Social Sciences (SPSS) for Windows Version 16.0 (SPSS Inc; Chicago, IL, USA) and EPI Info 3.5.1 Windows Version. The Z test and chi square test was used to observe the difference between different variables and strength of the relationship with logistic regression. p < 0.05 was considered as statistically significant. We calculated odds ratios (OR) and their 95% confidence intervals (95% CI). p < 0.05 was considered as statistically significant [19].

## Results

### Socio demographic background of schizophrenia

Among 210 schizophrenia patients, 78.6% were of younger age and less than 40 yrs, 61.9% male, 86.7% unemployed and 80.5% of them having monthly income less than 10000 NPR /month. As far religious background is concerned, most of the patients were Hindus 78.1% followed by Buddhist 15.2%, Christian 3.8% and Muslims 2.9%. However, the majority of patients of schizophrenia were from Dalit communities 26.2% followed by the Chettri 23.8%. It was also found that 46.2% of the patients were students, housewife 25.2% and others 8.6%, retired 6.7%, job holders 5.7% respectively (Table 1).

**Table 1:** Cross tabulation between socio demographic factors and schizophrenia in psychiatry inpatients

**Socio demographic factors**			**P Value**	** 95% CI**
Age	> 40 yrs	45(21.4)	0.0001†	[16.1, 27.6]
	<40 yrs	165(78.6)		[72.4, 83.9]
Gender	Female	80(38.1)	0.0001†	[31.5, 45.0]
	Male	130(61.9)		[55.0, 68.5]
Employment	Employed	28(13.3)	0.0001†	[9.0, 18.7]
	Unemployed	182(86.7)		[81.3, 91.0]
Monthly income	>10000/month	41(19.5)	0.0001†	[14.4, 25.5]
	<10000/month	169(80.5)		[74.5, 85.6]
Religion	Muslim	6(2.9)	-	[1.1, 6.1]
	Christian	8(3.8)		[1.7, 7.4]
	Buddhist	32(15.2)		[10.7, 20.8]
	Hindu	164(78.1)		[71.9, 83.5]
Ethinicity	Magar, Pun	11(5.2)	-	[2.6, 9.2]
	Others	17(8.1)		[4.8, 12.6]
	Newar	17(8.1)		[4.8, 12.6]
	Gurung	21(10)		[6.3, 14.9]
	Brahmin	39(18.6)		[13.6, 24.5]
	Chettri	50(23.8)		[18.2, 30.2]
	Dalit	55(26.2)		[20.4, 32.7]
Occupation	Labourer	8(3.8)	-	[1.7, 7.4]
	Farmer	8(3.8)		[1.7, 7.4]
	Job holder	12(5.7)		[3.0, 9.8]
	Retired	14(6.7)		[3.7, 10.9]
	Others	18(8.6)		[5.2, 13.2]
	Housewife	53(25.2)		[19.5, 31.7]
	Student	97(46.2)		[39.3, 53.2]

† p<0.01, statistically significant.

-P value cannot calculate.

### Schizophrenia and antipsychotic drugs

Data indicated that most of the schizophrenic patients had been receiving antipsychotics by trade names. Nearly 65.4% of schizophrenic male patient had received drugs by trade names which was found to be statistically significant p < 0.01. Though Nepal has essential list of drugs, most of the patients of schizophrenia had received drugs which are not listed there. An association was noticed between the ethnicity and the practice of using drugs from the National drug list of Nepal (p = 0.0001), it was revealed that drugs which were not included in the national drug list of Nepal were also commonly used in the Dalit 26%, Chettri 24.7%, Brahmin 12%, Gurung 12.7%, Newari 9.3%, Magar and Pun 4%, others 11.3% (Table 2).

**Table 2:** Cross tabulation between socio demographic factors, national drug list of Nepal, generic and trade names

**Socio demographic factors**		**National drug list of Nepal**		**Generic/trade**	
		**Essential drugs**	**Nonessential drugs**	**Generic**	**Trade**
Age	> 40 yrs	14 (23.3)	31 (20.7)	5 (20)	40 (21.6)
	<40 yrs	46(76.7)	119(79.3)	20(80)	145 (78.4)
	P Value		0.400×		0.545×
Gender	Female	21(35)	59(39.3)	16 (64)	64(34.6))
	Male	39(65)	91(60.7)	9(36)	121(65.4)
	P Value		0.336×		0.005†
Employment	Employed	10(16.7)	18(12)	3(12)	25(13.5)
	Unemployed	50(83.3)	132(88)	22(88)	160(86.5)
	P Value		0.246×		0.565×
Monthly income	>10000/month	13(21.7)	28(18.7)	7(28)	34(18.4)
	<10000/month	47(78.3)	122(81.3)	18(88)	151(81.6)
	P Value		0.375×		0.565×
Religion	Muslim	0 (0)	6 (4)	0 (0)	6(3.2)
	Christian	0(0)	8(5.3)	0(0)	8 (4.3)
	Buddhist	7(11.3)	25(16.7)	2(8)	30(16.2)
	Hindu	53(88.3)	111(74)	23(92)	141(76.2
	P Value		0.059×		0.312×
Ethnicity	Magar, Pun	5 (8.3)	6 (4)	2(8)	9 (4.9)
	Others	0(0)	17(11.3)	0(0)	17(9.2)
	Newar	3(5)	14(9.3)	3(12)	14(7.6)
	Gurung	2(3.3)	19(12.7)	0(0)	21(11.4)
	Brahmin	21(35)	18(12)	4(16)	35(18.9)
	Chettri	13(21.7)	37(24.7)	10(40)	40(21.6)
	Dalit	16(26.7)	39(26)	6(24)	49(26.5)
	P Value		0.0001†		0.154×
Occupation	Labour	5 (8.3)	3 (2)	0(0)	8 (4.3)
	Farmer	2(3.3)	6(4)	0(0)	8 (4.3)
	Jobholder	3(5)	9(6)	3(12)	9 (4.9)
	Retired	7(11.7)	7(4.7)	0(0)	14(7.6)
	Others	8(13.3)	10(6.7)	2(8)	16(8.6)
	Housewife	14(23.3)	39(26)	10(40)	43(23.2)
	Student	21(35)	76(50.7)	10(40)	87(47)
	P Value		0.053×		0.190×

† p<0.01, statistically significant.

× p>0.05, statistically not significant.

Table 3 shows cross tabulation information namely socio demographic factors, groups of antipsychotic drugs and the type of treatment given to the schizophrenic patients. Most of the patients received atypical antipsychotics among antipsychotics. There was an association among ethnicity, occupation and religion of the patients with the groups of antipsychotics prescribed. Data shows that 65.5% patients were Hindus followed by Buddhist 20.2, Christian 6.5% and Muslims 4.8% received atypical antipsychotic drugs. Ethnically, atypical antipsychotics drugs were consumed by 22.6% of Dalit patients, 26.6% Chettri patients, 9.7% Brahmin patients, 15.3% Gurung patients, Magar and Pun 4.8% and Newar 9.7%. Likewise, 48.4% of patients were students followed by housewife.

**Table 3:** Cross tabulation between socio demographic factors, groups of anti-psychotics, treatment given to the schizophrenic inpatient at the psychiatric ward

**Sociodemographic factors**		**Groups of drugs**			**Treatment**			
		**Miscellaneous**	**Typical antipsychotics**	**Atypical antipsychotics**	**ECT**	**Drugs and psychotherapy**	**Drug therapy**	**Combined therapy**
Age	>40 yrs	3(11.5)	14(23.3)	28(22.6)	2(100)	13(12.2)	27(36)	3(11.5)
	<40 yrs	23(88.5)	46(76.7)	96(77.4)	0(0)	94(87.9)	48(64)	23(88.5)
	P value	0.419×			0.0001†			
Gender	Female	21(80.8)	39(65)	70(56.5)	2(100)	44(41.1)	29(38.7)	5(15.4)
	Male	5(19.2)	21(35)	54(43.5)	0(0)	63(58.9)	46(1.3)	21(80.8)
	P value	0.057×			0.05×			
Employment	Employed	2(7.7)	10(16.7)	16(12.9)	0(0)	18(16.8)	8(10.7)	2(7.7)
	Unemployed	24(92.3)	50(83.3)	108(87.1)	2(100)	89(83.2)	67(89.3)	24(92.3)
	P value	0.519×			0.455×			
Monthly income	>10000/month	4(15.4)	13(21.7)	24(19.4)	2(100)	26(24.3)	9(12)	4(15.4)
	<10000/month	22(84.6)	47(78.3)	100(80.6)	0(0)	81(75.7)	66(88)	22(84.6)
	P value	0.794×			0.005 †			
Religion	Muslim	0(0)	0(0)	6(4.8)	0(0)	3(2.8)	3(4)	0(0)
	Christian	0(0)	0(0)	8(6.5)	0(0)	8(7.5)	0(0)	0(0)
	Buddist	0(0)	7(11.7)	25(20.2)	2(100)	21(19.6)	9(12)	0(0)
	Hindu	26(100)	53(88.3)	85(65.5)	0(0)	75(70.1)	63(84)	26(100)
	P value	0.003†			0.0001†			
Ethinicity	Magar, Pun	0(0)	5(8.3)	6(4.8)	2(100)	5(4.7)	4(5.3)	0(0)
	Others	3(11.5)	0(0)	14(11.3)	0(0)	9(8.4)	5(6.7)	3(11.5)
	Newar	2(7.7)	3(5)	12(9.7)	0(0)	8(7.5)	7(9.3)	2(7.7)
	Gurung	0(0)	2(3.3)	19(15.3)	0(0)	16(15)	5(6.7)	0(0)
	Brahmin	6(23.1)	21(35)	12(9.7)	0(0)	21(19.6)	12(16)	6(23.1)
	Chettri	4(15.4)	13(21.7)	33(26.6)	0(0)	26(24.3)	20(26.7)	4(15.4)
	Dalit	11(42.3)	16(28.6)	28(22.6)	0(0)	22(40)	22(40)	11(20)
	P value	0.0001 †			0.0001†			
Occupation	Labour	0(0)	5(8.3)	3(2.4)	0(0)	8(7.5)	0(0)	0(0)
	Farmer	2(7.7)	2(3.3)	4(3.2)	0(0)	4(3.7)	2(2.7)	2(7.7)
	Job holder	0(0)	3(5)	9(7.3)	0(0)	6(5.6)	6(8)	0(0)
	Retired	3(11.5)	7(11.7)	4(3.2)	0(0)	7(6.5)	4(5.3)	3(11.5)
	Others	2(7.7)	8(13.3)	8(6.5)	2(100)	2(1.9)	14(18.7)	2(7.7)
	Housewife	3(11.5)	14(23.3)	36(29)	0(0)	30(28)	18(24)	3(11.5)
	Students	16(61.5)	21(35)	60(48.4)	0(0)	50(46.7)	31(41.3)	16(61.5)
	P value	0.044 *			0.0006†			

†p<0.01, statistically significant.

* p<0.05, statistically significant.

× p>0.05, statistically not significant.

Schizophrenic patients received various types of treatment like ECT, drug and psychotherapy combined together. Most of the patients received a combination of drug therapy and psychotherapy. It was found that there was an association among different factors namely age, monthly income, religion and occupation and subsequent treatment given to the patients, which showed that 87.9% patients less than 40 years of age received a combination of psychotherapy and drug therapy whereas if the age of the patient was over 40 years they received drug therapy only. Religious background also indicates, there were 70.1% and19.6% Hindus and Buddhists respectively. Likewise, 40% Dalit were followed by Chettri 24.3% and Brahmin 19.6%. 46.7% were students followed by housewives 28% received a combination of both psychotherapy and drug therapy.

The most common antipsychotic drug prescribed was olanzapine. There was an association between the age of the patient and antipsychotics prescribed (p = 0.001). It was revealed if the age is <40 years, Olanzapine was prescribed to 84.7% patients but if the age of the patient is > 40 yrs. Risperidone was commonly prescribed 45.9% (p = 0.002) (Table 4).

**Table 4:** Coss tabulation between socio demographic factors and neuroleptics prescribed

**Socio demographic factors**		**Antipsychotics prescribed**							
		**Aripriprazole**	**Quetiapine**	**Clozapine**	**Risperidone**	**Haloperidol & Olanzapine**	**Haloperidol**	**Olanzapine**	**P Value**
		**N05AX12**	**N05AH04**	**N05AH02**	**N05AX08**	**-**	**N05AD01**	**N05AH03**	
	> 40 yrs	0(0)	0(0)	0(0)	17(45.9)	3(11.5)	14(23.3)	11(15.3)	0.002†
Age	<40 yrs	2(100)	5(100)	8(100	20(54.1)	23(88.5)	46(76.7)	61(84.7)	
	Female	0(0)	3(60)	3(37.5)	19(51.4)	5(19.2)	21(35)	29(40.3)	0.156×
Gender	Male	2(100)	2(40)	5(62.5)	18(48.6)	21(80.8)	39(65)	43(59.7)	
	Employed	0(0)	0(0)	2(25)	0(0)	2(7.7)	10(16.7)	14(19.4)	0.079×
Employment	Unemployed	2(100)	5(100)	6(75)	37(100)	24(92.3)	50(83.3)	58(80.6)	
	>10000/month	0(0)	0(0)	0(0)	9(24.3)	4(15.4)	13(21.7)	15(20.8)	0.580×
Monthly income	<10000/month	2(100)	5(100)	8(100)	28(75.7)	22(84.6)	47(78.3)	57(79.2)	
	Muslim	0(0)	0(0)	0(0)	0(0)	0(0)	0(0)	6(8.3)	0.001†
	Christian	0(0)	0(0)	0(0)	3(8.1)	0(0)	0(0)	5(6.9)	
	Buddhist	0(0)	2(40)	8(100)	10(27)	0(0)	7(11.7)	13(18.1)	
Religion	Hindu	2(100)	3(60)	0(0)	24(64.9)	26(100)	53(88.3)	48(66.7)	
	Magar, Pun	0(0)	2(40)	0(0)	0(0)	0(0)	5(8.3)	4(5.6)	0.001†
	Others	0(0)	0(0)	0(0)	3(8.1)	3(11.5)	0(0)	11(15.3)	
	Newar	0(0)	0(0)	0(0)	7(18.9)	2(7.7)	3(5)	5(6.9)	
	Gurung	0(0)	0(0)	0(0)	10(27)	0(0)	2(3.3)	9(12.5)	
	Brahmin	0(0)	0(0)	0(0)	2(5.4)	6(23.1)	21(35)	10(13.9)	
	Chettri	2(100)	3(60)	0(0)	6(16.2)	4(15.4)	13(21.7)	22(30.6)	
Ethinicity	Dalit	0(0)	0(0)	8(100)	9(24.3)	11(42.3)	16(26.7)	11(15.3)	
	Labourer	0(0)	0(0)	0(0)	0(0)	0(0)	5(8.3)	3(4.2)	0.12×
	Farmer	0(0)	0(0)	2(25)	0(0)	2(7,7)	2(3.3)	2(2.8)	
	Job holder	0(0)	0(0)	0(0)	0(0)	0(0)	3(5)	9(12.5)	
	Retired	0(0)	0(0)	0(0)	2(5.4)	3(11.5)	7(11.7)	2(2.8)	
	Others	0(0)	0(0)	0(0)	4(10.8)	2(7.7)	813.3)	4(5.6)	
	Housewife	0(0)	0(0)	0(0)	11(29.7)	3(11.5)	14(23.3)	25(34.7)	
Occupation	Student	2(100)	5(100)	6(75)	20(54.1)	16(61.5)	21(28.6)	27(37.5)	

† p<0.01, statistically significant.

× p>0.05, statistically not significant.

### Commonest antipsychotics prescribed

Olanzapine (Atypical Antipsychotics) was the commonest antipsychotic drug prescribed 34.3% followed by Haloperidol (Typical Antipsychotics) 28.6%, combination of haloperidol and olanzapine 12.4%, Risperidone 17.4%, clozapine 3.8%, Quetiapine 2.4% and Aripiprazole 1% (Atypical Antipsychotics ) respectively.

### Determinants of antipsychotics use by logistic regression

From the point of view of logistic regression analysis, it was found that Psychiatrist has a [OR 7.9, 95% (CI 1.209, 51.84)] more tendency of prescribing medicines not from the essential drug list Nepal if the patient is from Gurung community as compared to Magar and Pun. Psychiatrist has 6.0 and 4.6 times more tendency of prescribing medicines not from the essential drug list of Nepal among students [OR 6.0(95% (1.332, 27.324)] and housewives [OR 4.6(95% (0.979, 22.010)] respectively. It is also observed that the tendency of using antipsychotic drugs by trade names were [OR 3.3 (1.407, 8.031] times more among male patients as compared to female ones. All the findings were statistically significant (Table 5).

**Table 5:** Logistic regression table of non-essential drugs (drugs not included in the national list of essential drugs of Nepal), trade name and socio demographic factors

**Socio demographic factors**		**Nonessential drugs**	**Trade**
		**Odds ratio (confidence interval)**	**Odds ratio (confidence interval)**
	> 40 yrs	1	1
Age	<40 yrs	1.168(0.570, 2.393)×	0.906(0.320, 2.566) ×
	Female	1	1
Gender	Male	0.831(0.445, 1.549) ×	3.361(1.407, 8.031)†
	Employed	1	1
Employment	Unemployed	1.467 (0.634, 3.393) ×	0.873(0.243, 3.132) ×
	>10000/month	1	1
Monthly income	<10000/month	1.205 (0.576, 2.523) ×	1.727(0.669, 4.461) ×
	Muslim	1	1
	Christian	1	-
	Buddhist	-	-
Religion	Hindu	-	-
	Magar, Pun	1	1
	Others	-	-
	Newar	3.889 (0.695, 21.749) ×	-
	Gurung	7.917 (1.209, 51.841)*	-
	Brahmin	0.714 (0.186, 2.737) ×	-
	Chettri	2.372 (0.618, 9.099) ×	-
Ethnicity	Dalit	2.031 (0.542, 7.617) ×	-
	Labour	1	1
	Farmer	5.0(0.584, 42.797) ×	-
	Jobholder	5.0(0.720, 34.726) ×	1.037(0.144, 7.477) ×
	Retired	1.667(0.283, 9.822) ×	-
	Others	2.083(0.378, 11.482) ×	1.944(0.306, 12.350) ×
	Housewife	4.643(0.979, 22.010)*	0.889 (0.165, 4.777) ×
Occupation	Student	6.032(1.332, 27.324)*	1.815(0.315, 10.455) ×

†p<0.01, statistically significant.

* p<0.05, statistically significant.

× p>0.05, statistically not significant.

- P value cannot calculate.

## Discussion

### Socio-demographic details and schizophrenia

In the course of research, it was noticed that out of 210 cases of Schizophrenia, 58.76% of the cases involved males followed by 41.24% of females. The data shows that this finding resembles a research study conducted in Bangladesh as there also males suffer more with psychiatric disorder as compared to females [20]. However, this finding is different from a study done in Australia by Mant A et al. as it shows that Psychiatric illness is more common among female patients [21]. Most of the patients with Schizophrenia were below 40 years of age 78.6%, whereas only 21.4% of the patients were over 40 years. Similar nature of finding was reported by Maki P et al. that schizophrenia is commonly prevalent among the adolescence [22]. Most of the patients of Schizophrenia were Hindus 78.1%, followed by Buddhists 15.2%, Christians 3.8% and Muslims 2.9% respectively. This could be due to the fact that Nepal is Hindu dominated country and obviously most of the patients are expected to be from Hindu religion. Ethnically, most of the patients were Dalit 26.2% followed by Chettri 23.8%, Brahmin 18.6%, Gurung 10%, Newar 8.1%, Magar and Pun 5.2% and others 8.1%. The finding of present study is parallel to a study carried out by Kohrt BA in Nepal as the findings of the research stated that Psychiatric disorder is more common among Dalit/Nepali [23]. Schizophrenia was commonly seen in individuals having monthly income less than 10000 NPR /month (80.5%) and unemployed patients (86.7%). This finding is analogous to the outcome of study done in Sweden by Lessen E et al. as the data showed that utilization of psychotropic drugs were more among individuals with low income [24]. Similarly, different researched findings confirmed that schizophrenia appears to have socioeconomic and racial dimensions. It is most commonly seen amongst the poor in the United States, England, Japan, Norway, Ireland and Iceland, but in the context of India and possibly in Italy it is more frequently witnessed among the rich. The disease is also common among urban dwellers and blacks in the United States [25]. As far as occupation of the patients is concerned, Schizophrenia was most commonly seen among students 46.2% followed by housewives 25.2%, others 8.6% followed by retired persons 6.7%, jobholders 5.7%, farmers and labours 3.8%. This is similar to the conclusion of research done by Banerjee et al. as it revealed that psychiatric disorders in Nepal found among housewives and students [26,27]. In a study from Bangladesh also reported that psychiatric disorder is common among students and housewives [20].

### Drug utilization of antipsychotics

#### Drug therapy

In most of the cases of Schizophrenia, drug therapy and psychotherapy treatments were used together 51%; whereas in 35.7% cases were given either typical or atypical antipsychotics alone; in 12.4% cases a combination of multidrug were given comprising both typical and atypical antipsychotics and ECT was used only in 1% of the cases of schizophrenia. Psychotherapy was not used alone in none of the cases. It showed that psychotherapy along with drug therapy is more commonly used in schizophrenia management.

Most of the drugs were prescribed by trade names 88.1% whereas only 11.9% of the drugs were prescribed by generic name. This is similar to a study done in 2001 by PR Ravishankar on psychotropic drug utilization which also shows that 71.3% of the drugs were prescribed by trade names in Nepal [15]. In a different study done in the tertiary care hospital in Nepal indicates the low use of drugs by generic names in the past [28-30]. In MTH, the patient has to pay for the medications. Published evidence also has revealed by promoting the use of drugs by trade name will increase the cost of the therapy where other generic alternatives are available [31].

Antipsychotic drugs that have been included in the essential drug list of Nepal are Chlorpromazine, Fluphenazine, Haloperidol and Thioridazine [32]. Essential drugs were used in 28.6% Schizophrenia cases, which is quite similar to another study done by P.R Ravishankar on utilization of psychotropic drugs concluding that only 29.48% drugs were used from essential drug list of Nepal [15]. In a study done by Upadhyay in Nepal also shows that the prescribing drugs from essential drug list were low 37.09% [29]. This is a matter of concern and need to deal with by prescriber education, training campaigns are required to improve the therapeutic management of the patient and ensure better quality of life [15,33].

This study can serve as an outline for drug prescribing pattern and further research is required in this direction to improve the practice of prescribing pattern. Several other studies also shows that drug utilization study may be helpful for therapeutic audit and hypothesis generation for the improvement of prescribing pattern of drugs [34].

### Typical and atypical antipsychotics

As evident in the present study, most of the patients received atypical antipsychotics 59%, typical antipsychotics were used only in 28.6% of cases and in remaining cases a combination of typical and typical antipsychotics were used 12.4%. Olanzapine was the commonest antipsychotic drug being prescribed 34.3% followed by Haloperidol 28.6%, combination of haloperidol and olanzapine 12.4%, Risperidone 17.4%, Clozapine 3.8%, Quetiapine 2.4% and Aripiprazole 1%. The finding of this study is similar to the conclusion of research conducted by Dutta et al. at Uttaranchal, India in the year 2002–2003. His finding revealed that among the used antipsychotics, Olanzapine was prescribed most commonly 44.4% followed by Haloperidol 28.5%, Risperidone 9.5%, Quetiapine 6.3%, Aripiprazole5.2%, Ziprasidone, 4.2%, and Thioridazine 2.6% [35].

In a study done by Piparva KG et al. in India shows that the use of atypical antipsychotic drugs are used higher 43.83% than typical antipsychotic drug 26.32% [36]. Similar nature of studies carried out across the world also indicated the recent trend of use of atypical antipsychotics [37].

The published evidence confirms that atypical antipsychotics might be suitable for schizophrenic patients and better tolerated as compared to the typical antipsychotic drugs. The treatment pattern observed correlates with the world wide changing trends in the treatment of Schizophrenia [33].

In recent years, the induction of newer atypical antipsychotic agents like Olanzapine, Risperidone, Quetiapine and Ziprasidone have provided a better control of symptoms and reduced the chances of adverse effects especially the extrapyramidal ones in contrast with typical antipsychotic drugs. The newer atypical antipsychotics also proved to be better in improving negative symptoms, cognitive dysfunction and also efficacious in antipsychotic resistant schizophrenia [36].

Typical antipsychotics act through Dopaminergic pathway in the mesolimbic and mesofrontal pathway and are associated with extrapyrimidal adverse effects by blocking the D2 receptors in the brain. It is not seen with the atypical antipsychotics as they act through serotenergic pathways of brain. Atypical antipsychotics can also treat the negative symptoms during this disorder whereas the typical antipsychotics can only treat the positive symptoms [38,39]. Systematic review has shown that Olanzapine is more efficacious than other second generation antipsychotic drugs [40].

### Limitation

This research is based on the hospital study from Western Development Region of Nepal. A multi centric hospital based study with higher sample size will be beneficial to assess the current trend of antipsychotics all over Nepal.

## Conclusion

According to the utilization pattern of antipsychotics, it is concluded that atypical antipsychotics were used relatively more commonly than that of typical antipsychotics. Among the atypical antipsychotic drugs, there is a trend of using Olanzapine during Schizophrenia as compared to other atypical antipsychotic drugs in Western Nepal.

## Competing interests

The authors do not have any conflict of interest arising from the study.

## Authors’ contribution

IB designed the study, deduced the data, drafted the manuscript, and revised it. BR, PKC and IB2 planned the study with IB, acquired the data, conducted the data analysis, interpreted the data, and revised the manuscript. IB2 has also participated in the language editing along with IB and BR. BS participated in statistical analysis, interpreted the data, and revised the manuscript. PKS and ACS critically revised the manuscript. All the authors approved the final document.

## Authors’ information

Dr. Indrajit Banerjee MBBS, MD Pharmacology. Currently working as a Lecturer in the Department of Pharmacology, Manipal College of Medical Sciences, Pokhara, Nepal and Chief of Manipal Sanjeevani Clinic. He is the Chief Editor of International Journal of Physiology and Pharmacology (IJPP), Managing Editor of International Journal of Brain Research (IJBR), Section Editor of Nepal Journal of Epidemiology (NJE). He is an Organizing Committee member of International Epidemiological Association Conference 2013 and Confederation of Epidemiological Associations (CEA) Conference December 2013 Organized by Mahatma Gandhi University. He is also Assistant Secretary General of CEA. Mr. Bedanta Roy MSc Physiology. Currently working as Assistant Professor in the Department of Physiology, Manipal College of Medical Sciences, and Pokhara, Nepal. He is at present pursuing PhD in Neurophysiology. He is the Chief Editor of International Journal of Brain Research (IJBR), Managing editor of International Journal of Physiology and Pharmacology (IJPP), Section Editor of Nepal Journal of Epidemiology (NJE). He is an Organizing Committee member of International Epidemiological Association Conference 2013 and Confederation of Epidemiological Associations (CEA) Conference December 2013. He is also Assistant Secretary General of CEA.

Dr. Brijesh Sathian MD(AM), PhD. working as Assistant Professor and Bio-statistics Chief in the Department of Community Medicine, Manipal College of Medical Sciences, Pokhara, Nepal. He is the editorial board member of NJE, IJBS, AIIJMS, GMJ, IJPP, IJBR and Joint Organizing Secretory and treasurer of International Epidemiological Association Conference 2013. Joint Organizing Secretary CEA (Confederation of Epidemiological Associations) Conference December 2013 Organized by Mahatma Gandhi University. He is also the President of CEA.

Dr. P.K. Chakraborty MBBS, MD Psychiatry. Currently working as Professor in the Department of Psychiatry and Hospital Director Manipal Teaching Hospital, Pokhara, Nepal. He is an organizing committee member of International Epidemiological Association Conference 2013.

Dr. Archana Saha MBBS, MD Pharmacology. Currently working as Professor and Head in the Department of Pharmacology Manipal College of Medical Sciences, Pokhara, Nepal. She is the editorial board member of NJMS. She is organizing committee member of International Epidemiological Association Conference 2013.

Dr. Indraneel Banerjee, MBBS, MS, MRCS. Currently working as a Senior Resident, R. G. Kar Medical College, Kolkata, West Bengal, India.

## Pre-publication history

The pre-publication history for this paper can be accessed here:

http://www.biomedcentral.com/1471-244X/13/96/prepub
